# Enhancing electrochemical detection through machine learning-driven prediction for canine mammary tumor biomarker with green silver nanoparticles

**DOI:** 10.1007/s00216-024-05444-0

**Published:** 2024-07-20

**Authors:** Sinem Özlem Enginler, Tarık Küçükdeniz, Gamze Evkuran Dal, Funda Yıldırım, Gökçe Erdemir Cilasun, Fulya Üstün Alkan, Hazal Öztürk Gürgen, Nevin Taşaltın, Ahmet Sabuncu, Merve Yılmaz, Selcan Karakuş

**Affiliations:** 1 Department of Obstetrics and Gynecology, Faculty of Veterinary Medicine, Istanbul University-Cerrahpaşa, Avcılar, 34320 Istanbul, Turkey; 2 Department of Industrial Engineering, Faculty of Engineering, Istanbul University-Cerrahpaşa, Avcılar, 34320 Istanbul, Turkey; 3 Department of Pathology, Faculty of Veterinary Medicine, Istanbul University-Cerrahpaşa, Buyukcekmece, 34500 Istanbul, Turkey; 4 Department of Molecular Medicine, Aziz Sancar Institute of Experimental Medicine, Istanbul University, Fatih, 34104 Istanbul, Turkey; 5 Department of Pharmacology and Toxicology, Faculty of Veterinary Medicine, Istanbul University-Cerrahpaşa, Buyukcekmece, 34500 Istanbul, Turkey; 6 Department of Electrical and Electronics Engineering, Faculty of Engineering and Natural Sciences, Maltepe University, Maltepe, 34857 Istanbul, Turkey; 7 Department of Chemistry, Faculty of Engineering, Istanbul University-Cerrahpaşa, Avcılar, 34320 Istanbul, Turkey; 8 Health Biotechnology Center for Excellence Joint Practice and Research (SABIOTEK), Istanbul University-Cerrahpaşa, Istanbul, Turkey

**Keywords:** Biosensor, CA 15–3, MUC 1, Data science, Electrochemistry

## Abstract

This study developed an innovative biosensor strategy for the sensitive and selective detection of canine mammary tumor biomarkers, cancer antigen 15–3 (CA 15–3) and mucin 1 (MUC-1), integrating green silver nanoparticles (GAgNPs) with machine learning (ML) algorithms to achieve high diagnostic accuracy and potential for noninvasive early detection. The GAgNPs-enhanced electrochemical biosensor demonstrated selective detection of CA 15–3 in serum and MUC-1 in tissue homogenates, with limits of detection (LODs) of 0.07 and 0.11 U mL^−1^, respectively. The nanoscale dimensions of the GAgNPs endowed them with electrochemically active surface areas, facilitating sensitive biomarker detection. Experimental studies targeted CA 15–3 and MUC-1 biomarkers in clinical samples, and the biosensor exhibited ease of use and good selectivity. Furthermore, ML algorithms were employed to analyze the electrochemical data and predict biomarker concentrations, enhancing the diagnostic accuracy. The Random Forest algorithm achieved 98% accuracy in tumor presence prediction, while an Artificial Neural Network attained 76% accuracy in CA 15–3-based tumor grade classification. The integration of ML techniques with the GAgNPs-based biosensor offers a promising approach for noninvasive, accurate, and early detection of canine mammary tumors, potentially revolutionizing veterinary diagnostics. This multilayered strategy, combining eco-friendly nanomaterials, electrochemical sensing, and ML algorithms, holds significant potential for advancing both biomedical research and clinical practice in the field of canine mammary tumor diagnostics.

**Graphical Abstract:**

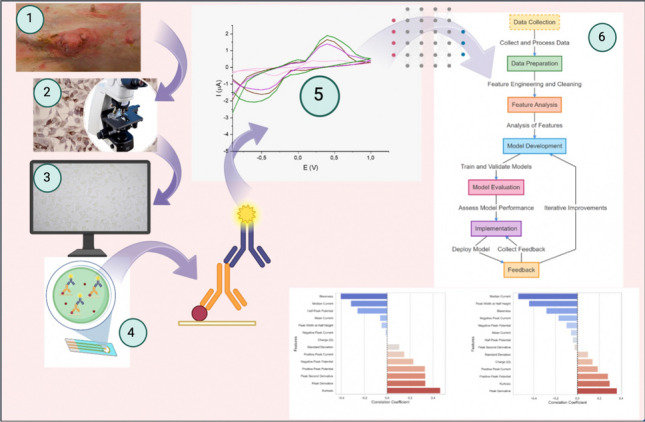

**Supplementary Information:**

The online version contains supplementary material available at 10.1007/s00216-024-05444-0.

## Introduction

Canine mammary tumors are primarily observed in elderly dogs, especially after 6 years of age [1, 2]. The prevalence of malignant lesions in canine mammary tumors ranges from 26 to 73% [3–5]. As canine mammary tumors share similarities with human breast cancer, understanding its mechanism is crucial for preventing human breast cancer progression. Additionally, biomarkers used for diagnosis, treatment options, and prognosis determination are common to both [6]. Biomarkers are proteins that express characteristics specific to each cancer cell and are usually measurable in the blood or other tissues (such as tumor tissue). They can provide valuable information about the presence of a disease, treatment outcomes, and patient prognosis. When these proteins known as tumor-associated antigen are found in other tissues, serum, or urine at quantities that differ from those of healthy individuals, they serve as biomarkers. Malignant transformation is thought to be the cause of their increasing production [7–9]. Integrins, immunoglobulin-like particles, cadherins, cancer antigen 15–3 (CA 15–3), carcinoembryonic antigen (CEA), and selectins are biomarkers involved in intracellular adhesion [10]. The mammary gland associated-antigen MUC1, also known as episialin, polymorphic epithelial mucin, can be assessed through CA 15–3 [11]. CA 15–3 (in serum), also expressed as mucin 1 or MUC1 (in tissue), is overexpressed in tumoral cells and enters the bloodstream [12]. Detecting these biomarkers in tissue, blood, or various other body fluids aids in early diagnosis, tumor grading, prognosis, and monitoring of response to treatment in bitches with mammary tumors [13]. CA 15–3 was determined using a variety of methods, such as immunosorbent enzyme-linked assays, chemiluminescence immunoassays, enzyme immunoassay, radioimmunoassay (RIA), and electrochemical and photoelectrochemical immunoassays [14]. However, because of their long process, difficult label collection, and other errors, the approaches are unable to address the growing clinical need for the rapid detection of CA 15–3. Furthermore, conventional techniques might not be able to detect tumor markers at incredibly low concentrations, which emphasizes the necessity of developing novel nanoscale strategies.

By incorporating these small size and exceptional morphology, nanostructures play a pivotal role in advancing cancer diagnosis and treatment. With the assistance of nanomaterials, electrochemical biosensors can traverse biological barriers and reach specific human tissues and cells. Biosensors leverage the precise electrochemical performance of nanosensors by integrating a biologically derived recognition element into a physico-chemical converter [15–20]. This combination holds the potential for exciting developments in the field of cancer research enhancing the accuracy and efficiency of early-stage cancer detection and monitoring. When compared to traditional analytical methods such as immunohistochemistry or ELISA, biosensors are remarkably easy to use and reliably produce findings, partly due to their small particle sizes (lab-on-a-chip devices) [18].

Machine learning (ML) is frequently employed as a rapid method for training machines and developing predictive algorithms that facilitate efficient decision-making in data science [21, 22]. The evaluation of tumor size and grade by ML contributes to the determination of mammary tumor type and aids in early diagnosis. ML computational models stand as the most effective techniques in biomedical fields and data science for obtaining favorable clinical data for prediction and classification of target problems. The application of ML in tumor detection and the prediction of tumor presence or absence has been proposed to advance breast tumor research. Additionally, ML approaches may be utilized to predict the size and severity of a tumor. Traditionally, disease diagnosis relies on human observation to identify specific signal features. In conditions such as tumor, where early detection is vital, the development of computer-aided diagnostic approaches has accelerated in data science. These methods aim to transform qualitative diagnostic criteria into quantitative features, thereby enhancing classification accuracy. In a review, Teng et al. presented innovative biosensor developments for detecting prostate tumor, highlighting the utilization of both single and multiple biomarkers. The potential of ML to improve biosensor performance metrics in prostate tumor diagnosis was also underscored [20]. In another study, Pennacchio et al. proposed a bacterial biosensor that utilizes gold nanoparticles (AuNPs) modified with a hydrophobin chimera combined with ML algorithms and a simplified process for immobilizing antimicrobial peptides, which enables cost-effective measurement of bacterial contamination [18]. By integrating ML algorithms with an NP-based biosensor, clinical data can be analyzed intelligently and accurately, leading to improved identification of tissue and serum sample biomarkers that are both selective and sensitive. In addition to enhancing in sensitivity and accuracy of sophisticated sensor applications, this technique supports real-time analysis and intelligent evaluation. The integration of ML algorithms holds the potential to transform several industries, including biotechnology, point-of-care services, health monitoring, and early disease diagnosis due to its innovative capabilities.

The novelty of this study lies in its multifaceted approach to biosensor development and biomarker detection in biomedical and sensor applications. Firstly, the utilization of GAgNPs provides an eco-friendly and cost-effective alternative in the field of canine mammary tumor sensors. These nanoparticles were instrumental in developing a unique electrode modification, facilitating the creation of a sensitive impedimetric immunosensor capable of quantitatively detecting canine mammary tumor biomarkers, specifically CA 15–3 and MUC-1, in serum and tissue homogenate samples. Additionally, the antibodies coated on the GAgNPs improved the sensor’s sensitivity and selectivity of the sensor by enhancing the coating on the working electrode. With a broad linear concentration range of 5 to 100 U mL^−1^, the biosensor demonstrated selective detection of CA 15–3 and MUC-1, boasting respective LODs of 0.07 and 0.11 U mL^−1^ within a 60-s voltammetric cycle. Furthermore, the sensor exhibited ease of use and good selectivity in detecting CA 15–3 and MUC-1 in clinical canine serum and tissue homogenate samples, underscoring its efficacy in diagnosing canine mammary tumors. Secondly, the integration of ML techniques enhances the biosensor’s predictive capabilities, enabling accurate predictions of biomarker concentrations for CA 15–3 and MUC-1. Notably, ML predictions further enhanced the diagnostic accuracy. Overall, this innovative biosensor strategy holds significant promise for advancing of veterinary diagnostics.

## Material and method

### Materials

The powdered maca root, purchased from Arioğlu Company (Türkiye), has the following composition are as follows protein (14.30), water (6.28), ash (3.22), and oil (1.31). Moreover, it has 1.25 mg g^−1^ of glucosinolate, 0.16 mg g^−1^ of maca amide, and 0.18 mg g^−1^ of alkaloid. Sodium hydroxide (NaOH) was purchased from Merck Company (Germany). Whatman® qualitative filter paper (diam. 25 mm) and silver nitrate (AgNO_3_, purity ≥ 99.0%) were obtained from Sigma-Aldrich Company Ltd. (USA). All samples were filtered using a polyvinylidene difluoride (PVDF) membrane and a sterile syringe filter with a 0.45-µm retention. All chemicals and reagents were used as received without further purification.

### Animal material

Ethical approval for the study was obtained from the Istanbul University-Cerrahpaşa, Veterinary Faculty Ethics Committee Unit (2021/55, 16.11.2021). Bitches brought to our clinic with malignant mammary tumors were included into the study (*n* = 17), they underwent bilateral mastectomy for the treatment of mammary tumors, and during this operation, superficial inguinal lymph nodes at both sites were also extirpated. After the operation, the extirpated tissues were sent to pathology for histopathological examination and to evaluate of lymph node metastasis. Prior to surgery, 5 mL of blood samples were collected from each bitch. One gram of mammary tissue samples from the suspected tumorous lesions was obtained postoperatively. At the owner’s request, a 4-year-old spayed Cocker Spaniel bitch underwent a preventive (prophylactic) bilateral mastectomy, resulting in the acquisition of a normal mammary gland from the right inguinal mammary lobe. Upon palpation of all mammary glands, no tumoral lesions were detected, and the control group (*n* = 1) had no prior history of endocrine or mammary disorders. Additionally, a 5-mL blood sample was taken from this bitch before surgery. The determination that there of no size variation in the axillary and popliteal lymph nodes in all bitches included in the study was confirmed by palpation. To investigate the presence of lung metastasis, three projections of thorax radiography (left lateral to right lateral (Li-Ld), right lateral to left lateral (Ld-Li), and ventrodorsal (VD)) were taken from all the bitches with mammary tumors in the study. A complete blood count and chemistry panel were among the laboratory tests performed on all the bitches in the study to detect any unusual changes that could affect serum CA 15–3 concentration (hepatic and kidney diseases) and bilateral mastectomy operation. Only the bitches with blood parameters within the reference ranges were included in the study. All blood samples were centrifuged at 3500 rpm for 20 min, and the extracted serum samples were stored at − 86 °C until use. A gram of mammary tissue samples (g) was defatted in the laboratory and homogenized (Miccra D-1, ART, moderne Labortechnik e.K., Germany) in 4 mL of PBS (pH 7.2). Then, this mixture was centrifuged at 3500 rpm for 20 min at 4 °C. All supernatants were collected and stored in centrifuge tubes (1.5 mL) at − 86 °C until use. After collection, all tissue homogenates and serum samples were stored in a cold chain for electrochemical biosensor applications. Additionally, tissue samples from each animal underwent pathological examination for histopathological analysis and immunohistochemical determination of the MUC-1 biomarker.

### Histopathology and immunohistochemistry

Tissue samples taken for histopathological examination and immunohistochemical labeling were fixed in 10% neutral buffered formalin solution for 48 h. Routine tissue follow-up procedures were performed, and paraffin blocks were prepared. The sections were cut at a thickness of 4 µm and stained with dyes (hematoxylin and eosin), and histopathological evaluation was performed under a light microscope (Nikon Eclipse Ci-L). The tumor samples were diagnosed and graded histopathologically according to Goldschmidt et al. [5] and Pena et al. [23], respectively.

Each case was labeled with MUC-1 antibody using the immunohistochemistry (IHC) technique. Sections, 4-µm thick, were taken on positively charged glass slides and deparaffinized by incubation in a dry oven at 65 °C, followed by immersion in xylene, and then passed through graded ethyl alcohol concentrations from high to low (100%, 96%, 85%, and 70%, respectively) and rehydrated in distilled water. They were then washed three times with 0.1 M phosphate buffer solution (PBS; pH 7.4). To block endogenous peroxidase activity in the tissues, the sections were incubated in 0.3% H_2_O_2_ in methanol for 20 min at room temperature. Subsequently, the sections were heat-treated in a microwave oven for 20 min in pH 6.0 citrate buffer solution. Protein blocking was performed using the serum cocktail included in the IHC staining kit (Thermo Scientific IHC kit Cat. No.TP-125-HL). The polyclonal rabbit anti-MUC-1 antibody (Ab Clonal, Cat. No. A0333) was diluted to 1/100 and incubated at 37 °C for 90 min. After the primary antibody incubation, the sections were washed with PBS. The secondary antibody and HRP solution were applied for 10 min each. Following additional washes, the reaction was completed with 3,3′-diaminobenzidine (DAB; Thermo Scientific Cat. No. TA-125-HD). The sections were then incubated for 5 min in Mayer’s hematoxylin for background staining and covered with coverslips. The stained preparations were evaluated based on the extent and intensity of the staining. Immunohistochemical staining was evaluated using a light microscope, and MUC-1 staining reactions were scored as follows: no staining (0), weak (1), moderate (2), and intense (3) based on the extent and intensity of staining [24]. All microscopic examinations were independently evaluated by two pathologists, and the final scores were determined by comparing the results. Digital micrographs were obtained from H&E- and IHC stained preparations with at Nikon Eclipse Ci-L microscope and a Nikon DSQi2 camera system. All images were enhanced using Macromedia Fireworks 8, at the same settings.

### Cell culture

To check the sensitivity of the sensor, canine mammary carcinoma cell line CMT-U27 was used as a positive control. Chemicals used in the cell culture were products of Gibco (Invitrogen, CA, USA). CMT-U27 cells were maintained in Dulbecco’s Modified Eagle Medium/Nutrient Mixture F-12 supplemented with 1% L-glutamine, 1% penicillin–streptomycin solution, and 10% fetal bovine serum in a humidified atmosphere containing 5% CO_2_ at 37 °C. The culture medium was changed every 2 days. When cells reached 80–90% confluence, a 0.25% trypsin/EDTA solution was added to remove adherent cells from the surface. For immunocytochemistry, 1 × 10^5^ cells/well were seeded on coverslips in 24-well plates and incubated overnight.

### Immunocytochemistry

To demonstrate MUC-1 expression in the CMT-U27 cell line, the medium was aspirated with the pipette over cells grown on coverslips in 24-well plates. Without allowing the cells to dry, 1000 μL each of methanol cooled at -20°C was added, and the cells were incubated at -20°C and fixed under this condition for 20 minutes. Methanol was aspirated from the wells, followed by immediate washing three times with PBS (pH 7.4). After incubation in 0.3% H_2_O_2_ prepared in methanol for 10 min at room temperature, the wells were washed three times with PBS, and coverslips were removed from the wells and transferred onto parafilm. The coverslips were then incubated with the protein block solution in the IHC kit for 15 min, followed by incubation with MUC-1 antibody diluted 1/200 for 60 min at room temperature. After incubation, coverslips were washed again with PBS. The secondary antibody and then horseradish peroxidase were applied for linking. The reaction was completed with DAB. Mayer Hematoxylin was performed for background staining.

### Statistical analysis

The statistical analysis was conducted to assess the relationships between clinical variables and tumor grades, aiming to enhance the understanding of their interdependencies and impact on cancer diagnostics. Spearman’s rank correlation coefficient was used as the primary method for this analysis. This choice is due to its non-parametric nature, making it suitable for not-perfectly normally distributed data and ordinal variables. The dataset comprised 17 entries, with variables including “Location,” “Size,” “Lymph Node Metastasis,” “Lung Metastasis,” “MUC-1 Expression.” Each of these variables was assessed for its association with tumor grade. Spearman’s correlation was selected for its ability to measure the strength and direction of association between ranked variables.

### Preparation of electrochemical biosensor

In our previous study, we detailed the synthesis of GAgNPs via a cost-effective ultrasonication method at room temperature [25]. The prepared GAgNPs were produced at a reasonable cost by using ultrasonication at 25 °C. To create the green solution, maca root powder was cleaned, dried in a vacuum oven at 70 °C, combined with distilled water, and allowed to sit at 25 °C in the dark for 5 days. Following filtration through a sterile syringe filter with a micron size of 0.45, 0.42 g of AgNO_3_ and 0.1 g of NaOH were dissolved in 250 mL and 125 mL of distilled water, respectively. After adding the NaOH solution dropwise to the silver solution, the mixture was sonicated for half an hour at room temperature. Ultimately, a sterile syringe filter with a 0.45 µm was used to filter the final nanostructure.

In this study, electrochemical tests were performed at 25 ± 1 °C using the Ebtro Voltammetric Electrochemical workstation (Türkiye). Gold electrodes (Ebtro instruments, Türkiye) with a surface area of 2 cm^2^ were used as working electrodes, and alternating current voltammograms were recorded from − 0.05 to − 0.45 V and a scanning rate of 50 mV s^−1^ at 25 °C. The electrodes were equilibrated in PBS (pH 7.4) until a stable current peak was observed. Before the single layer was formed, the gold electrodes were cleaned with DI H_2_O, polished with a 0.1-μm diamond solution (Buehler, Lake Bluff, IL), and sonicated for around 5 min in a low power sonicator to get rid of bound particles. After that, the working electrodes underwent a series of oxidation and reduction cycles in 0.5 M H_2_SO_4_ between − 0.3 and 1.5 V to electrochemically wash them. For the Ag NP-based sensors, following the cleaning process, the Au electrodes were rinsed with deionized water, dried using N_2_ gas, and modified with the nanoformulation. To prepare the biosensors, we applied 10 μL of GAgNPs solution onto a gold-coated electrode and dried it in an oven at 50 °C for 5 min. Following this, the GAgNPs-coated sensor was left to rest at room temperature for 15 min. For electrochemical measurements, we treated the GAgNPs-coated sensor with 3 μL of antibody solution (Ab1), and it stored in the refrigerator for 1 h. Subsequently, we washed the electrode surface with PBS 7.4 solution to remove any unbonded capture antibody and obtain the Ab1/GAgNPs-coated sensor. As a control, the sensor was prepared using the same procedure, except without immobilizing Ab1. To detect CA 15–3 (4.9 mg mL^−1^, MYBioSource) and MUC-1 (0.5 mg mL^−1^, Biolegend), 10 µL of MUC-1 and CA 15–3 antibodies (concentration ranging from 500, 2000, 50,000, and 100,000 cells mL^−1^ in 50 mM PBS buffer) were dropped onto the prepared Ab1/Ag NPs-coated sensor and incubated for 30 min at room temperature.

### Electrochemical analysis of clinical serum and tissue homogenate samples

The clinical serum samples obtained from bitches for this study were supplied by the Istanbul University-Cerrahpaşa, Veterinary Faculty, Department of Obstetrics and Gynecology clinic (Table 1). All the collected blood samples were centrifuged at 2000 × g for 10 min and were subsequently diluted tenfold with PBS solution (“Animal material" section). These serum samples were collected from various bitches before the surgical procedure. Prior to the electrochemical analysis, serum samples were prepared following the procedure described earlier (“Histopathology and immunohistochemistry” section). Subsequently, various concentrations of CA 15–3 (500, 2000, 50,000, and 100,000 cells mL^−1^) were dissolved in tenfold diluted serum for the electrochemical analysis. Following this preparation step, measurements were taken by dripping 10 µL of CA 15–3/MUC-1 protein-based solutions (pH 7.4), serum samples, and tissue homogenates onto the sensor surface.

### Machine learning-based prediction of tumor grade using electrochemical data

In this research, an extensive application of ML algorithms was applied to enhance the prediction of tumor grades based on cyclic voltammetry (CV) data from canine mammary tumors. The dataset was based on CV measurements derived from canine serum and tissue homogenate samples. These measurements were crucial for determining the concentrations of the specific biomarkers, CA 15–3 and MUC-1, associated with the presence of mammary tumors. The initial phase of the analysis involved a feature engineering process designed to transform raw CV data into a structured format suitable for ML algorithms. Features crafted from the CV readings were aimed at capturing the complex behavior of the electrochemical responses associated with canine mammary tumor biomarkers, CA 15–3 and MUC-1. From the raw CV data, several features were derived that encapsulate both the basic and complex characteristics of the electrochemical curves.

- **Charge (Q)**: Represents the total charge involved in the electrochemical reaction, integral to understanding the overall activity level of the biomarker.
- **Half-peak potential**: Indicates the potential at which the current is half of its peak value, providing insights into electrochemical kinetics.
- **Peak width at half height**: Measures the width of the peak at its half-maximum, related to the dispersion of the signal and indicative of the reaction uniformity.
- **Mean current and median current**: These statistical measures give an average level of current during the CV measurement, reflecting the general behavior of the biomarker’s electrochemical response.
- **Standard deviation, kurtosis, and skewness**: These features describe the distribution of current readings, offering details on the variability, tailedness, and asymmetry of the electrochemical signal, respectively.
- **Peak derivative and peak second derivative**: Reflect the first and second rates of change of the current, important for identifying subtle shifts in the electrochemical response.
- **Positive and negative peak currents**: Indicate the maximum upward and downward peaks, respectively, providing a direct measure of the biomarker’s highest and lowest electrochemical activities.
- **Positive and negative peak potentials**: The potentials at which the maximum and minimum currents occur are crucial for understanding the electrochemical properties of the biomarker.

Following the feature engineering, a correlation analysis among the engineered features was performed. This step was crucial to identify the dependencies and relationships among the variables, which could influence the subsequent ML models. Feature importance was assessed using the Random Forest algorithm. This approach was employed to determine which features that significantly influence the predictive models. The importance assigned to each feature guided the selection process for the most relevant variables, thereby ensuring that the models focused on the most impactful data. ML algorithms were then tested for their ability to distinguish between tumor and non-tumor samples. This classification performance testing was critical to validate the effectiveness of the algorithms in making accurate diagnostic predictions. Separate classification models were developed for CA 15–3 and MUC-1 samples to classify the tumor grade. Each biomarker was analyzed using a tailored set of ML algorithms to accurately predict the severity and grade of the tumors. A variety of ML models have been explored for their effectiveness in predicting tumor presence, and grades, including Ensemble Methods such as Random Forest, XGBoost, and LightGBM, were known for their robustness against overfitting and ability to handle complex datasets, as well as Artificial Neural Networks (ANN), which were employed for their superior pattern recognition capabilities to model complex interactions within the electrochemical data.

A leave-one-out cross-validation strategy was employed to train the models, ensuring their generalizability of the models and mitigating overfitting. The rigor of this method, where each sample in the dataset is used once as the test set, while the remaining samples from the training set allows for a thorough assessment of the model performance.

The effectiveness of each model was evaluated using various metrics. The accuracy, defined as ${\text{Accuracy}}=\frac{TP+TN}{TP+TN+FP+FN}$, measures the overall correctness of the model. Precision, calculated as ${\text{Precision}}=\frac{TP}{TP+FP}$, assesses the model’s ability to identify only relevant instances as positive. Recall, or sensitivity, quantified by ${\text{Recall}}=\frac{TP}{TP+FN}$, evaluates the model’s ability to identify all actual positives. The F1-score, which is the harmonic mean of precision and recall, is given by $\text{F1-score}=2\cdot \frac{{\text{Precision}}\times {\text{Recall}}}{{\text{Precision}}+{\text{Recall}}}$, providing a balance between precision and recall in cases of uneven class distributions. These metrics collectively furnished a comprehensive assessment of each model’s capacity to classify the tumor grades accurately.

Post-validation, the models were integrated into a diagnostic framework that utilizes real-time CV data to predict tumor grades. This integration was intended to markedly enhance both the accuracy and timeliness of tumor classification, thereby improving the effectiveness of treatment planning.

**Fig. 1:**
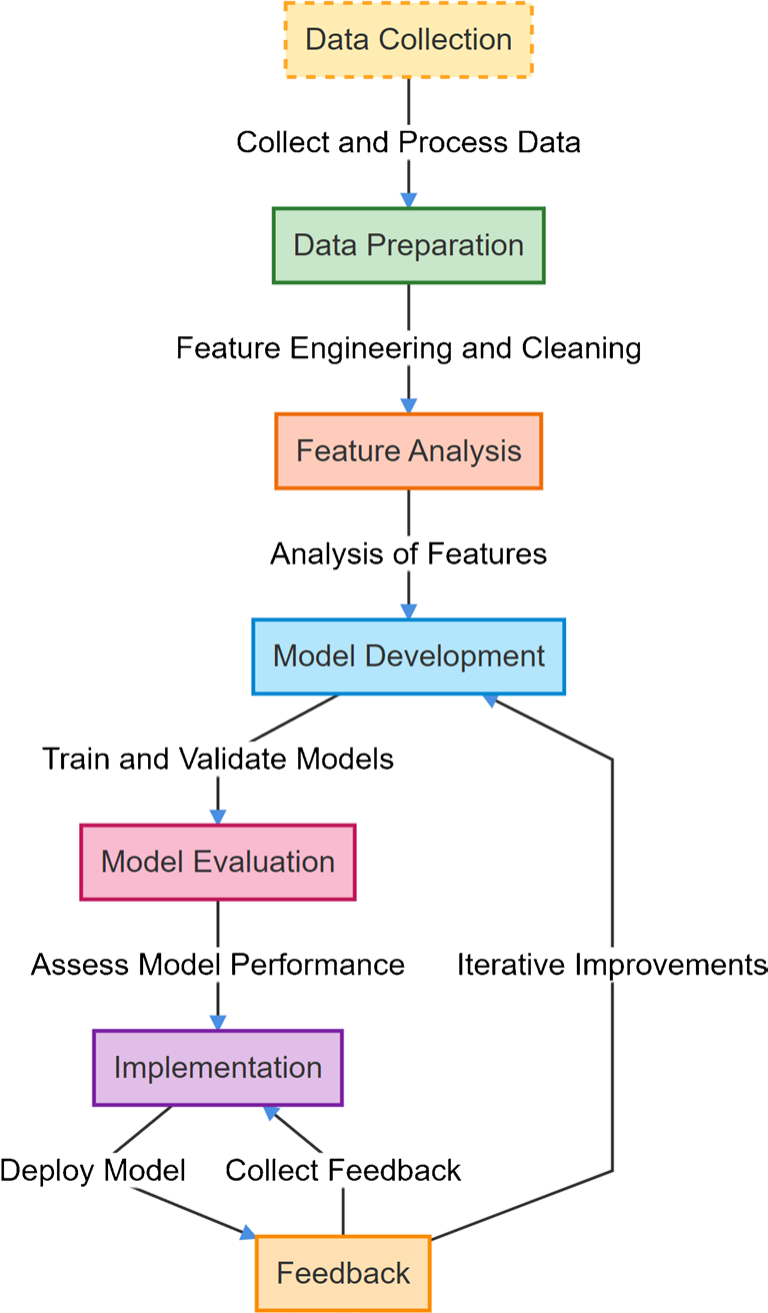
Flowchart of ML models for enhanced detection of canine mammary tumor biomarkers

The entire process, from data collection through the sensor to the final classification of tumor grades, is detailed in a flow chart included in this section (Fig. 1). This visual representation aids in understanding the sequential steps involved in the methodology, highlighting the logical progression from raw data processing to detailed ML analysis.

## Results and discussion

### Clinical findings

**Fig. 2:**
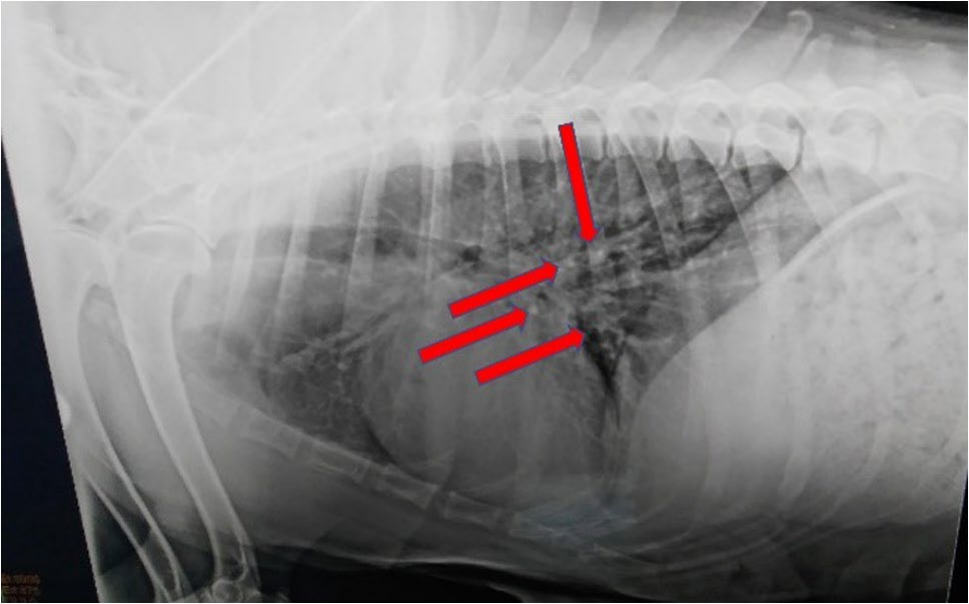
Lung metastases (red arrows) due to malignant mammary tumor in 11-year-old mix breed bitch in this study

In this study, 17 bitches were between 8 and 13 years of age (median age, 11.17 ± 1.59 years). The involved bitches’ mean weights were noted as 17.52 ± 8.17 kg which were suffering from malignant mammary tumors in this study. Control bitch (*n* = 1) was 4 years old and weighed 20 kg. The bitches were from different breeds, such as Mix (*n* = 6), Italian Greyhound (*n* = 1), Border Terrier (*n* = 2), Boxer (*n* = 1), American Cocker Spaniel (*n* = 3), Golden Retriever (*n* = 2), Yorkshire Terrier (*n* = 1), and Cavalier King Charles Spaniel (*n* = 1), and control bitch’s (*n* = 1) breed was Cocker Spaniel in this study. During the clinical inspection, the location of the mammary lobes where the primary malignant mammary tumor development was detected as follows: nine of them were detected in the inguinal lobes (M5), five in the caudoabdominal (M4), and three in the cranioabdominal (M3) mammary lobes. After X-ray evaluation, distant lung metastasis was detected in two of these animals (Fig. 2). All clinical details are shown in Table 1.

**Table 1:** Clinical data of the cases

No	Age (year)	Breed	Body weight (kg)	Primary mammary tumor location/staging
1	13	Mix	20	R5/ T1N0M0
2	13	Italian Greyhound	30	R3/ T1N0M0
3	10	Mix	17	R4/T2N1M0
4	12	Border Terrier	12	L3/ T1N0M0
5	12	Boxer	27	L5/ T2N0M0
6	12	American Cocker Spaniel	12	L5/ T2N1M1
7	11	Mix	23	R5/ T1N0M0
8	8	Golden Retriever	27	L4/ T1N0M0
9	11	Mix	25	L5/ T2N1M1
10	13	Golden Retriever	28	L4/ T1N0M0
11	10	Mix	12	R5/ T1N0M0
12	11	Yorkshire Terrier	6	L4/ T1N1M0
13	8	American Cocker Spaniel	12	R5/ T2N0M0
14	11	Cavalier King Charles Spaniel	8	L4/ T2N1M0
15	13	Border Terrier	7	L5/ T1N0M0
16	10	American Cocker Spaniel	22	R5/ T1N1M0
17	12	Mix	10	R3/ T1N0M0
Control 1	4	Cocker Spaniel	20	R5/-

3, cranioabdominal; 4, caudoabdominal; 5, inguinal. *R* right, *L* left, *T* tumor size, *N* lymph node metastasis, *M* distant metastasis (lung)

Sorenmo et al. reported that the occurrence of malignant mammary tumors can be increased in elderly bitches with the range of 7–13 years, which is in line with our findings (11.17 ± 1.59 years) [26, 27]. Edmunds et al. [28] reported a higher incidence of mammary tumor formation according to the breed distribution as Setter, American Bulldog, Spaniel, Akita, Yorkshire Terrier, and Cocker Spaniel. In this study, the mix breed (*n* = 6) is found to be the most predominant breed to malignant mammary tumor formation. Since carcinomas are epithelial tumors, they metastasize to regional lymph nodes and distant organs, primarily to the lungs via the lymphatics [29]. Sarcomas are mesenchymal tumors and metastasize directly to the lungs via the bloodstream. The most active mammary lobe in terms of tumor development in dogs is the 3rd cranioabdominal, 4th caudoabdominal, and 5th inguinal mammary lobes [30–32]. Metastasis of tumors to these lymphatics is also very important. While the caudoabdominal and inguinal mammary lobes drain into the superficial inguinal lymph node, the cranioabdominal mammary lobes drain mostly into the axillary lymph node and secondarily into the superficial inguinal lymph node; so, the tumors tend to metastasize to these lymph nodes firstly if they are of epithelial origin [33, 34]. A link has been determined between malignant canine mammary tumors and tumor location previously. Mammary tumors generally occur in the 3rd, 4th, and 5th lobes. It has been determined that mammary tumors in these lobes have more malignant characteristics than those in other lobes [35, 36]. This study found a statistically significant difference (*P* < 0.05) between the histological malignancy degree of mammary tumors and their location, in line with the literature. However, histological grading was found to increase as the tumor size increased. In addition, in line with the results stated in that study reported by Yamagami et al., the prognosis of bitches was also worsened, and lymph nodes and distant metastases can be occurred [37]. In this study, a statistically significant difference (*P* < 0.01) was found between the histological grade and the size of the tumor. In addition, there is a positive relationship between the degree of histological malignancy and lymph node/lung metastases and the age of the bitches in this study. It has been determined that the risk of mammary tumor development increases with age in bitches [38]. Grading the tumor not only gives the clinician important information regarding treatment but also allows the clinician to have information about the follow-ups required throughout the animal’s life. Grading of the tumors is based on some parameters such as tubule formation, nuclear atypia, and mitotic count [39]. Karayannopoulou et al. reported that grade III tumors have a poor prognosis as they can spread to lymph nodes than grade I and II tumor types [40]. Additionally, sarcomas can be more aggressive than carcinomas in bitches [39].

### Histopathological findings

**Fig. 3:**
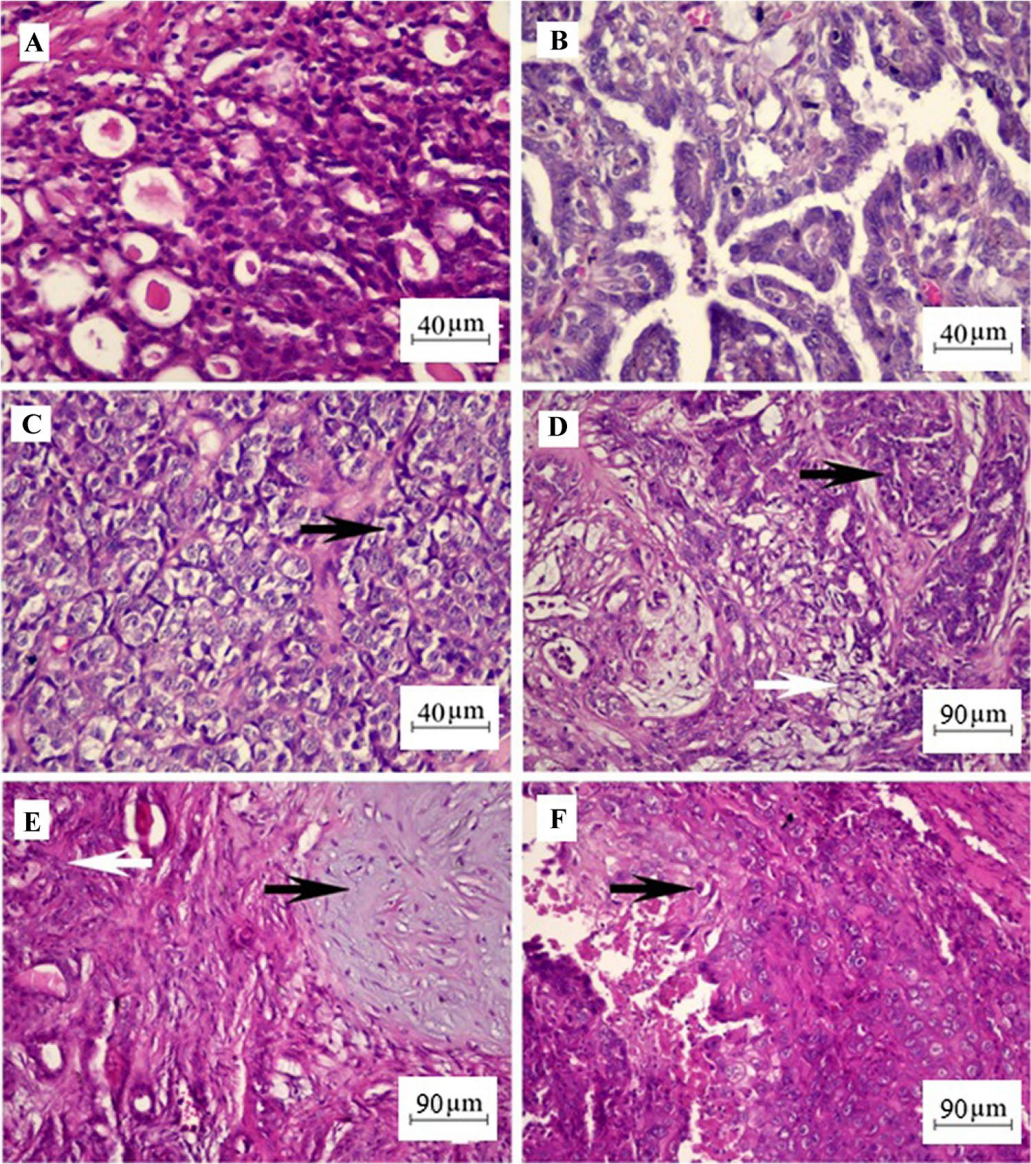
**A** Carcinoma-simple, tubular structures. **B** Carcinoma-simple, tubulopapillary structures.** C** A group of neoplastic cells in a carcinoma-solid showing mitotic figures (arrow). **D** A carcinoma-complex type including the luminal epithelium (black arrow) and myoepithelial component (white arrow). **E** A carcinoma-mixed type showing mesenchymal (black arrow) and epithelial (white arrow) proliferations in the same area. **F** An adenosquamous carcinoma including keratinized cells (arrow). Bar = 40 µm (**A**, **B**, **C**), and 90 µm (**D**, **E**, **F**); H&E staining

**Fig. 4:**
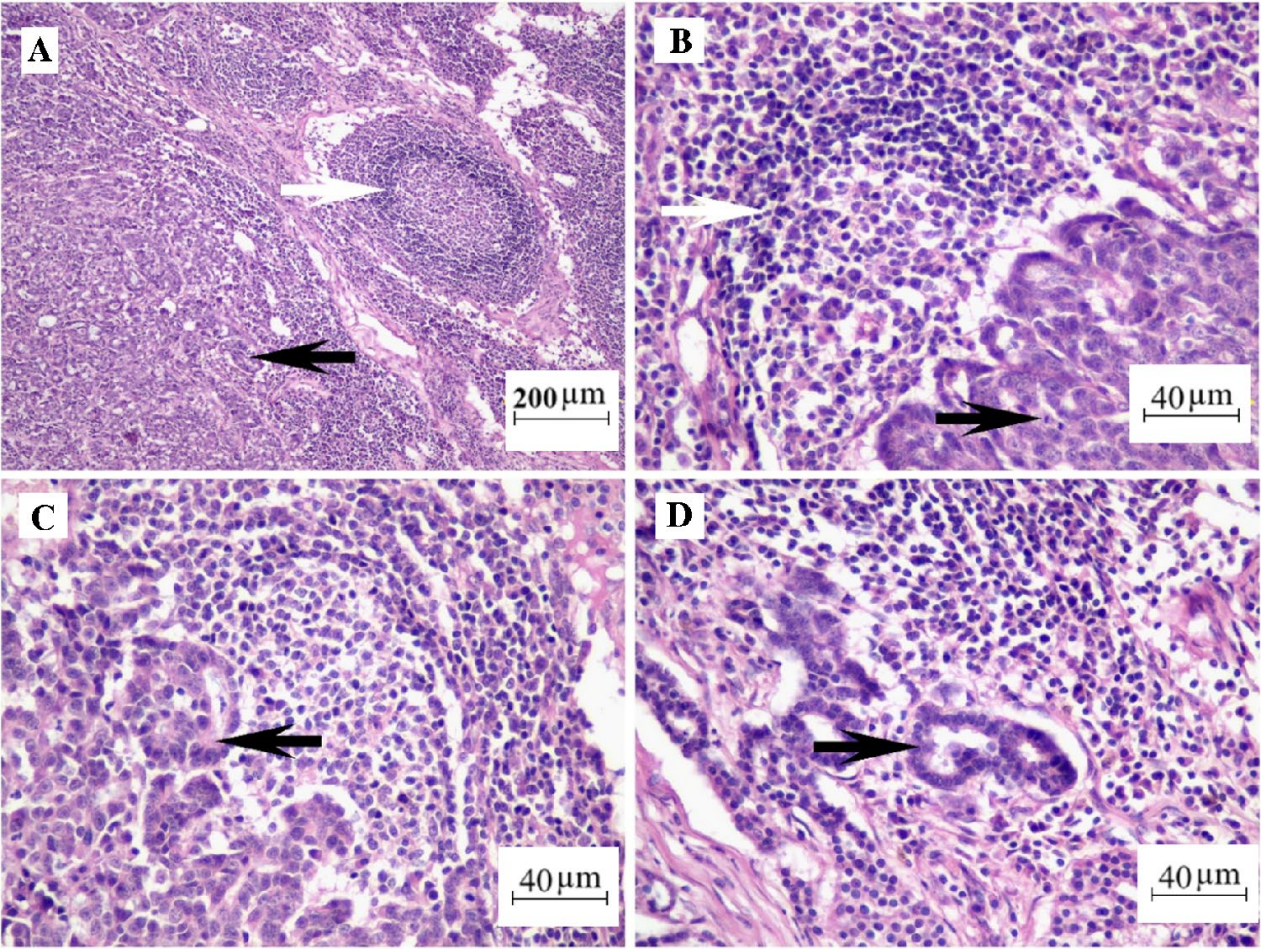
**A** Lymph node metastasis in a carcinoma-simple-tubular-type case, tubular structures (black arrow), reactive lymph follicle (white arrow). **B** An occupied lymph follicle (white arrow) by neoplastic tubular structures and a mitotic figure (black arrow) showing the activity of the tumor.** C** A group of neoplastic cells are arranged in a tubular pattern (black arrows). **D** Individual neoplastic tubules scattering in the lymph nodes (black arrow). Bar = 200 µm (**A**) and 40 µm (**B**, **C**, **D**); H&E staining

Sixteen of the tumors were histopathologically evaluated as carcinoma and one as sarcoma. The histological subtypes and their corresponding numbers of tumors were as follows: intraductal papillary carcinoma (*n* = 1), carcinoma with a simple-tubular or tubulopapillary pattern (*n* = 6), solid carcinoma (*n* = 2), complex-type carcinoma (*n* = 3), mixed-type carcinoma (*n* = 3), sarcoma (*n* = 1), and adenosquamous carcinoma (*n* = 1). The case of intraductal papillary carcinoma showed a stratified population of duct epithelial cells with malignancy-related characteristics. Carcinomas with a tubulopapillary pattern or simple tubulary were composed of neoplastic cells, predominantly cuboidal epithelial cells with eosinophilic cytoplasm in tubular or tubulopapillary morphologies (Fig. 3A, B). These cells had large oval vesicular nuclei and showed marked nuclear pleomorphism, hyperchromaticity, and abnormal nuclear structures. Two cases were diagnosed as carcinoma-solid type, characterized by tumoral cell clusters with numerous mitotic figures (Fig. 3C). The carcinoma-complex type included luminal and myoepithelial neoplastic cells arranged in the tubules and solid groups (Fig. 3D). Mesenchymal and epithelial neoplastic components were involved in the carcinoma-mixed type (Fig. 3E). One case was diagnosed as sarcoma, while the other was diagnosed as adenosquamous carcinoma. Adenosquamous cell carcinomas were identified by islands and cords of epithelial cells containing keratin filaments with the formation of keratin pearls (Fig. 3F). Microscopically, tumor metastasis was detected in the superficial inguinal lymph nodes in six of a total of 17 cases (Fig. 4). Four of these cases were simple carcinoma, one was a complex carcinoma, and the other was a sarcoma with lung involvement. All details such as tumor types, grades, and metastasis situations related to the cases are summarized in Table 2. Canine mammary tumors can include epithelial (adenoma or carcinoma), mesenchymal (sarcomas), or rarely myoepithelial cells (benign mixed tumors or carcinosarcoma), combined or separately [41]. Although studies have shown that the types of malignant dog mammary tumors are different in most countries, most of these tumor types have been found to be carcinomas. This study obtained a result compatible with this literature [41].

### Immunohistochemistry findings

**Fig. 5:**
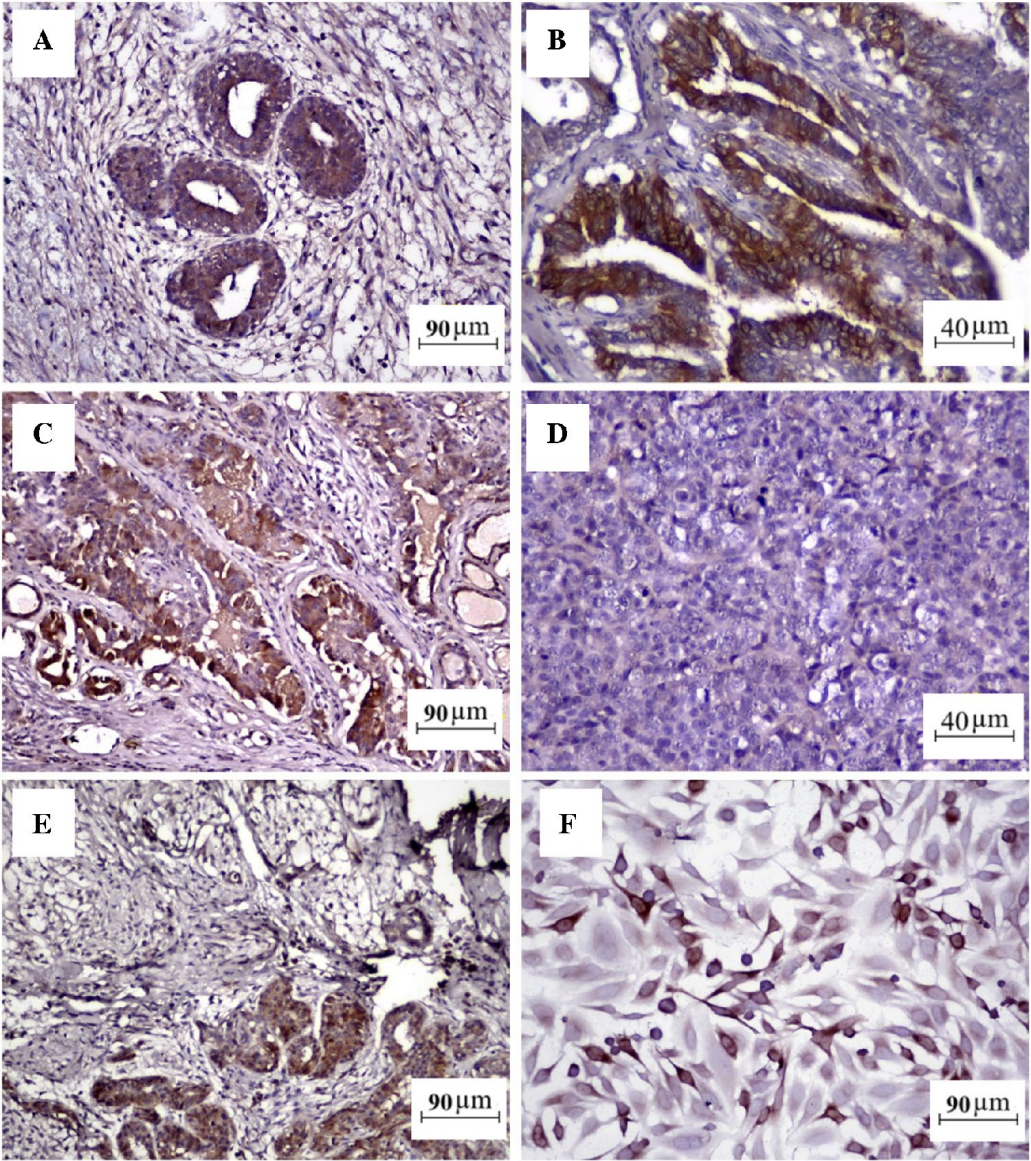
**A** A hyperplastic area shows slight positivity. **B** and** C** Strong positivity of a carcinoma-simple (tubulopapillary (**B**) and tubular (**C**)). **D** Negative staining in carcinoma-solid type. **E** Strong MUC-1 expression in only in the luminal epithelium part of a carcinoma-mixed type. **F** CMT U27 cells show strong MUC-1 expression in immunocytochemistry staining. Scale bar = 90 µm (**A**, **C**, **E**, **F**) and 40 µm (**B**, **D**); DAB chromogen for labeling and Mayer Hematoxylin background staining

MUC-1 labeling was detected predominantly in the luminal epithelium of the tumors at various levels. A tissue sample was obtained from an animal without a tumoral story to compare MUC-1 expression between tumoral tissues and normal mammary glands. Histologically, the sample exhibited mild hyperplastic and fibrous reactions. Immunohistochemically, benign hyperplastic areas demonstrated a slightly positive reaction, especially on the apical surfaces of the luminal epithelial cells (Fig. 5A), whereas the neoplastic epithelium showed strong positivity, especially in carcinoma-simple (tubulopapillary) types (Fig. 5B and C). One of the carcinoma-solid cases showed negative MUC-1 expression (Fig. 5D), whereas the other displayed slight positivity. The immunohistochemical reactions of the carcinoma-complex type and mixed-type tumors were comparable. In both tumor types, only the luminal epithelium was positive (Fig. 5E). CMT-U27 cells exhibited strong positivity for the MUC-1 antibody in immunocytochemistry staining (Fig. 5F), and the immunolabeling results are shown in Table 2. However, MUC1 antibodies were higher in grade III tumors, indicating tumor aggressiveness [42]. An important finding of this study is the expression of MUC-1 in benign canine mammary gland lesions. The reaction was mostly observed in the apical membrane of epithelial cells and extended to the cytoplasm of the luminal epithelial cells. This result is in absolute agreement with those observed in human and canine mammary tissues [43, 44]. When we evaluated the distribution of the positivity of the MUC-1 among mammary tumor cases, 11 of the 17 tumors were stained with different intensities. The MUC-1 positive staining rate was 54.5%. Manuali et al. stated that they observed MUC1 activity in 68% of the analyzed cases that displayed strong immunostaining in a mixed pattern (involving the entire cell surface and the cytoplasm), which comprises the whole cell population in several carcinoma samples. These findings are similar to those of our study. According to previous studies, a higher expression of MUC-1 is detected in more metastatic cancers [45]. In this study, six metastases were detected in superficial inguinal lymph node (SILN) metastases positive. However, only one metastatic case showed strong positivity for MUC-1, whereas the others were negative for MUC-1 expression. One of these metastatic cases was a sarcoma. This can be explained by the negative MUC-1 expression. The other four metastatic mammary tumor cases conflicted with the previous data. However, there was no meaningful relationship between MUC-1 expression and the type and grade of the tumors in our study. Manuali et al. showed that CA 15–3 has a positive correlation with tumor grade (higher CA 15–3 serum concentrations were observed in grades II and III than in grade I carcinoma types). Additionally, high levels of CA 15–3 were correlated with poor clinical stage and a poor prognosis. Apart from that, the study confirmed that tumor size, skin ulceration, necrosis, inflammation, and histological type of mammary cancer have no relation to serum levels of CA 15–3 [44]. This controversy can be explained by the histologically heterogeneous structures of canine mammary tumors. The CMT-U27 (ductal invasive carcinoma) cell line is a cell line consists of a single cell type showing epithelial or myoepithelial cell morphology isolated from the mammary gland of a 14-year-old female dog. It expresses low levels of cytokeratin, vimentin, and smooth muscle actin (SMA) filaments. The CMT-U27 cell line expresses 25% progesterone but not estrogen [46]. The cell line is examined for studies of mammary carcinomas, comparative in vitro studies of mammary tumors, and preclinical testing of drugs in veterinary oncology [47]. In this study, CMT-U27 cells were used as positive control to verify the efficacy of the CA-15–3 probe. First, an immunocytochemical staining was performed to show MUC-1 expression in CMT-U27 cells. The cells were labeled strongly with the MUC-1 antibody, as in a previous study showing MUC-1 positivity in CMT-U27 cells [44]. Campos et al. have investigated the level of MUC1 in tissue and CA-15–3 in serum of dogs with mammary gland cancer additionally they have investigated how the two markers correlate with the evolution of disease [48]. They have reported that the increased concentrations of MUC1 in the serum of dogs with mammary cancer are associated with the presence of metastasis to regional lymph nodes [48]. In some of our cases, an increased MUC-1 was observed in animals with metastasis, and this strong immunopositivity with MUC-1 correlated with the advance of disease. These results indicate that determining the expression of MUC-1 by IHC helps to predict the malignancy of tumor in dogs with a mammary tumor. In literature, it is reported that CA 15–3 is the most preferred serum biomarker in breast cancer patients with its low sensitive properties, and the detection of CA 15–3 in the diagnosis of primary breast cancer is insufficient. For this reason, it is used in monitoring approaches, especially when other markers such as CEA are detected at the same time. In veterinary medicine, the published studies were made based on a small number of heterogeneous patients. Therefore, more studies need to be done before it can be considered as an adequate biomarker [49]. In this study, we did not identify any significant correlation between tumor type, tumor grade, metastasis status, and MUC-1 expression, in which is consistent with the findings of Kazsak (2018) [49]. We attribute these results to the heterogeneous nature of canine mammary tumors. Additionally, our investigation determined that MUC-1 expression does not yield effective results as a biomarker in tissue samples. Furthermore, our study suggests that evaluating MUC-1 expression by immunohistochemistry is not a very suitable technique for canine mammary tumors.

The histopathological diagnosis and MUC-1 expression in IHC were summarized in Table 2 in this study.

**Table 2:** Histopathological diagnosis and immunohistochemical data

No	Diagnosis	Grade	SILN* metastases findings	MUC-1 expression
1	Carcinoma-solid type	III	-	0
2	Carcinoma-mixed type	I	-	1
3	Carcinoma-simple (tubular)	II	+	0
4	Carcinoma-mixed type	II	-	1
5	Adenosquamous carcinoma	III	-	0
6	Sarcoma	III	+	0
7	Carcinoma-complex type	II	-	1
8	Carcinoma-simple (tubulopapillary)	II	-	2
9	Carcinoma-simple (tubulopapillary)	III	+	0
10	Carcinoma-complex type	II	-	1
11	Carcinoma-simple (tubulopapillary)	II	-	1
12	Carcinoma-complex type	II	+	1
13	Carcinoma-solid type	III	-	1
14	Carcinoma-simple (tubulopapillary)	III	+	3
15	Carcinoma-simple (tubular)	III	-	1
16	Intraductal papillary carcinoma	I	+	0
17	Carcinoma-mixed type	II	-	3
Control 1	Mild hyperplastic and fibrous reactions	–-	––-	2

*SILN superficial inguinal lymph node

### Statistical analysis of clinical, histopathological and immunohistochemical data in predicting tumor grade

The Spearman’s correlation coefficients and their corresponding *P*-values were calculated for each variable against tumor grade. The results are summarized in Table 3.

**Table 3:** Correlation coefficients and significance levels

Parameter	Correlation coefficient	*P*-value	Significance
Location	0.512	0.036	+
Size	0.622	0.008	+ +
Lymph node metastasis	0.069	0.792	NS
Lung metastasis	0.410	0.102	NS
MUC-1 expression	− 0.193	0.457	NS

NS: *P* > 0.05, *P < 0.05, **P < 0.01

The analysis revealed that “Location” and “Size” are significantly correlated with tumor grade, with *P*-values indicating strong statistical significance (below 0.05). This suggests that these variables are important predictors of the severity of tumor and could be vital in clinical assessments and further research into tumor grading. Conversely, variables such as “Lymph Node Metastasis,” “Lung Metastasis,” “MUC-1 Expression” did not show significant correlations with tumor grade. The higher *P*-values suggest that there is insufficient evidence to conclude a strong association with tumor grade, indicating that they may be less critical in predicting tumor severity within this dataset.

### Electrochemical results

In this study, we conducted electrochemical measurements on the electrodes to track the stepwise alteration of the electrode and the changes in the sensing interface features after modification with GAgNPs. This analyze aimed to determine the concentrations of CA 15–3 and MUC-1 biomarkers in blood serum and tissue homogenate samples, respectively. Figure 5 illustrates the sensitive and selective detection capabilities of the GAgNPs-coated sensor, presenting comparative results from the measurements. For CA 15–3, assessments were conducted on blood serum samples, demonstrating the Ab1/GAgNPs-coated sensor’s efficacy in detecting and quantifying biomarker within a biological matrix commonly used in clinical diagnostics. Similarly, the sensor’s performance in detecting MUC-1 from tissue homogenates underscores its potential for tissue-based diagnostics, offering valuable insights into the tumor microenvironment. According to the electrochemical results presented in Fig. S1, a noticeable increase in the redox peak current for the biosensor is observed with rising concentrations of biomarkers. As both the tumor grade and biomarker (CA 15–3 and MUC-1) concentrations increase, the Ab1/GAgNPs-coated sensor redox peaks at higher values. This enhancement can be attributed to the larger active area and higher electronic conductivity of the GAgNPs, which facilitate the accelerated interfacial electron transfer.

The combination of nanomaterials with electrochemical biosensors offers the potential to at once monitor several cancer signals. These devices are highly beneficial in the early diagnosis of malignancies and are distinguished by their rapidity, simple use, and affordability. In 2018, Pacheco et al. developed a molecularly imprinted polymer (MIP)-based electrochemical sensor for monitoring breast cancer. The sensor was created by directly imprinting CA 15–3 onto a screen-printed gold electrode (Au-SPE). A linear relationship was established between the peak current intensity of the redox probe and the logarithm of the CA 15–3 concentration within the range of 5 to 50 U mL^−1^, achieving a LOD of 1.5 U mL^−1^ [50]. In 2023, AlGhamdi et al. synthesized a Fe–gallic acid MOF embedded in an epoxy methyl cellulose polymer thin film. With a sensitivity range of 0.05–570 U mL^−1^, they created this to identify CA 15–3 in the serum of breast cancer patients. In comparison to existing detection techniques, this affordable and reliable probe demonstrated a LOD of 0.01 U mL^−1^ [14]. Oliveira et al. developed a printed electrode on paper using carbon nanotube ink, graphite pencil, and silver nanoparticles (AgNPs) ink. In this study, they modified the electrode with gold nanoparticles (AuNPs) and a MIP to target CA 15–3. Despite shortcomings in saliva samples, the CNE/AuNP/MIP sensor showed promise for detecting CA 15–3 levels in serum [51]. Despite recent advances, these methods are still in their infancy, and further studies are required to offer real-time and in-situ sensing abilities. Comparing the suggested biosensor to counterparts listed in Table 3, it has a greater linear range, a remarkable LOD for CA 15–3 detection and is simpler and easier to fabricate. Advanced nanomaterial techniques, frequently involving hazardous chemicals and labor-intensive preparation processes of the high prices and energy consumption, are the foundation of many investigations listed in Table 3. By using a green nanomaterial-based detecting method and needing less preparation time, our sensor is more economical. Using the Ab1/GAgNPs-coated biosensor, specific interaction between anti-CA 15–3 antibody and CA 15–3 antigen results in the formation of an immunocomplex. Using a redox mechanism, this technique allows for indirect CA 15–3 detection. The binding of CA 15–3 to the recognition sites of the Ab1/GAgNPs-coated sensor is essential for the biosensor to work. The current signal is correlated with the amount of CA 15–3 because this binding event masks the probe’s electrode transfer and reduces the electrochemical response (see Table 4). The experimental findings confirmed that CA 15–3 and MUC-1 electrochemical immunosensors were successfully constructed and can sense substances. We sought to determine if antibody-antigen (Ab-Ag) binding was responsible for the observed alterations in the electrochemical signal using experimental investigations with control groups and different analytes. It was discovered that the current signal in the control group and with other analytes remained mostly constant by analyzing the CV graphs of the experimental group (electrode modified with Ab) and the control group (electrode without Ab) using the ML technique. Targeting, however, the biomarkers in the experimental group resulted in a notable change in the current signal. These experimental results clearly show that the immunological sensor is sensitive and selective.

**Table 4:** Comparison of various sensors for determination of CA 15–3 tumor biomarkers

Sensor	Results	Ref
Molecularly imprinted polymer-based electrochemical sensor	CA 15–3 concentration within the range of 5 to 50 U mL^−1^, achieving a LOD of 1.5 U mL^−1^	[50]
Fe–gallic acid MOF embedded in an epoxy methyl cellulose polymer thin film	With a sensitivity range of 0.05–570 U mL^−1^, they created this to identify CA 15–3 in the serum of breast cancer patients. LOD: 0.01 U mL^−1^	[14]
Carbon nanotube/gold nanoparticles	A sensitivity of 0.013936 μA/U mL^−1^, LOD of 1.16 U mL^−1^ and quantification of 3.87 U mL^−1^	[51]
O-Phenylenediamine on Au screen-printed electrodes	CA 15–3 concentration range of 0.25 to 10.00 U mL^−1^ and a LOD of 0.05 U mL^−1^	[52]
Mesoporous silica films modified electrode	CA 15–3 concentration range of 0.1 mU mL^−1^ to 100 mU mL^−1^ with a LOD of 9 μU mL^−1^ for electrochemiluminescence mode, and 10 mU mL^−1^ to 200 U mL^−1^ with a LOD of 5.4 mU mL^−1^ for electrochemistry mode	[53]
Ab1/GAgNPs-coated biosensor	CA 15–3 and MUC-1 with respective LOD of 0.07 and 0.11 U mL^−1^	This study

**Fig. 6:**
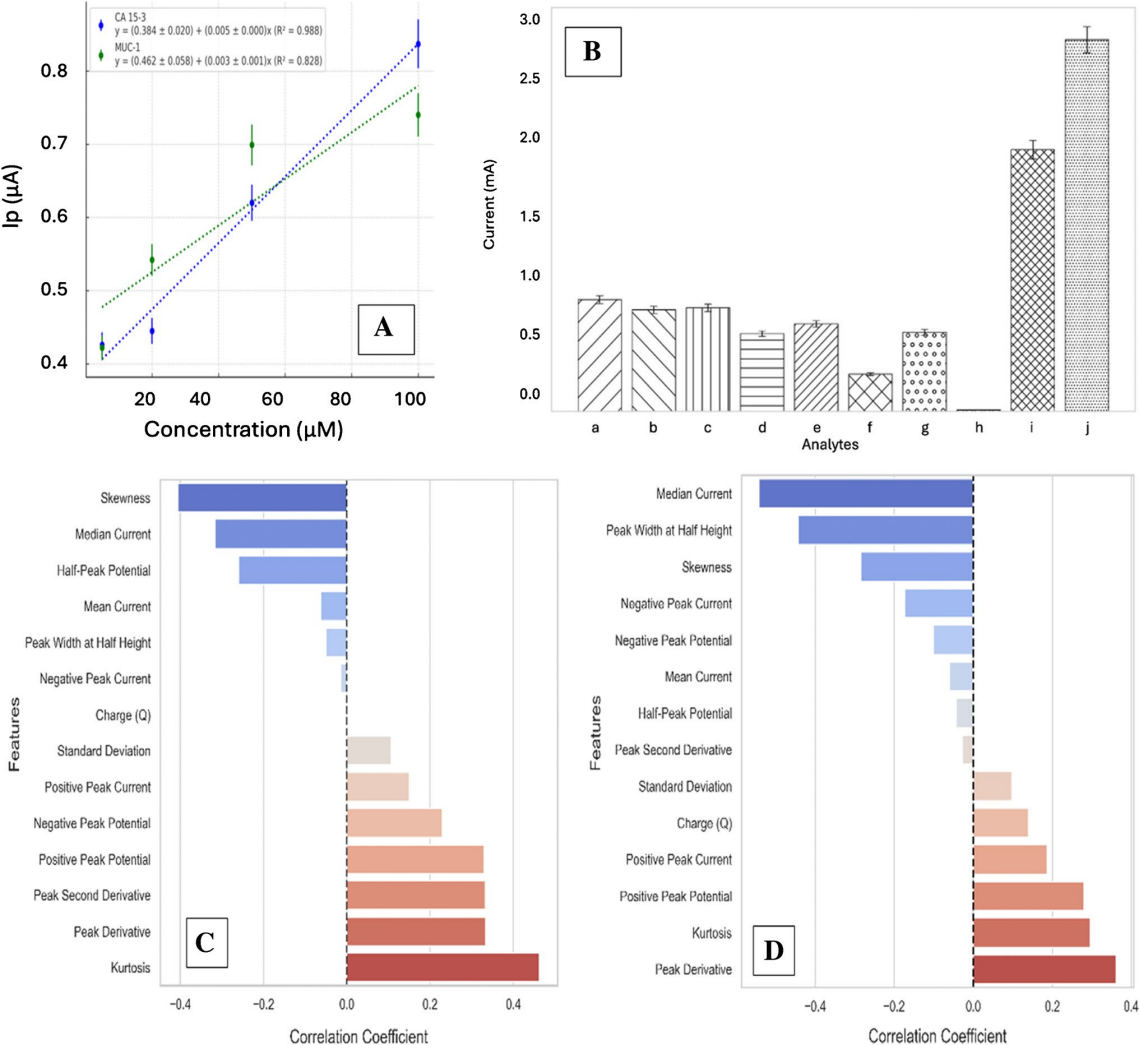
**A** Sensitivity. **B** Selectivity of immunosensor for (**a**) fructose (12 mM), (**b**) galactose (12 mM), (**c**) glucose (12 mM), (**d**) lactose (12 mM), (**e**) maltose (12 mM), (**f**) oxytocin (10 IU mL^−1^), (**g**) progesterone (2.2 mg mL^−1^), (**h**) prostaglandin (250 µg mL^−1^), (**i**) CA 15–3 at 100 U mL^−1^, (**j**) MUC-1 at a concentration of 100 U mL.^−1^. **C–D** Correlation coefficients of CV-derived features with canine mammary tumor grade for biomarkers CA 15–3 (**C**) and MUC-1 (**D**)

The performance characteristics of the immunosensor for the various analytes are depicted in Fig. 6. The sensitivity of the immunosensor is illustrated in Fig. 6A, demonstrating the detectability of target analytes, including CA 15–3 and MUC-1. The biosensor exhibits selective detection of CA 15–3 and MUC-1, with LOD of 0.070 and 0.112 μM, respectively, alongside a high sensitivity of 0.0045 and 0.0031 μA/cm^2^ mM, respectively, as shown in Fig. 6A. Figure 6B showcases the immunosensor’s selectivity, wherein diverse analytes, such as fructose (12 mM), galactose (12 mM), glucose (12 mM), lactose (12 mM), maltose (12 mM), oxytocin (10 IU mL^−1^), progesterone (2.2 mg mL^−1^), prostaglandin (250 µg mL^−1^), CA 15–3 (100 U mL^−1^), and MUC-1 (100 U mL^−1^). This thorough experimental research demonstrates the immunosensor’s selectivity identifying biomarkers. The distinct responses of the immunosensor to CA 15–3 and MUC-1 at specific doses emphasize its selectivity towards these target biomarkers. In this study, the selectivity analysis for various biomarkers and compounds revealed that CA 15–3 exhibited the highest selectivity value of 2.843, demonstrating excellent specificity in its detection within the tested set. Similarly, MUC-1 also showed substantial selectivity with a value of 2.002, indicating its effective differentiation from other substances. Additionally, Fig. 6C and D presents the correlation coefficients between CV-derived characteristics and the canine mammary tumor grade for CA 15–3 and MUC-1, respectively. These coefficients offer crucial insights into the relationship between the electrochemical characteristics and severity of canine mammary tumors, aiding in diagnosis and disease tracking.

### Machine learning-based prediction of tumor grade using CV data

This section presents the findings from the application of ML algorithms to predict tumor presence and grade in canine samples using differential pulse voltammetry data. A correlation analysis was conducted to refine the feature set and enhance the accuracy of the models by analyzing the relationship between which is the engineered features and tumor grades for both CA 15–3 and MUC-1 biomarkers. This analysis, summarized in Table 5 and illustrated in the correlation chart (Fig. 6C–D), was instrumental in identifying the features that were most significantly relate to the severity of the tumor, thereby guiding the selection of features for subsequent modeling. For CA 15–3, features such as kurtosis (0.462), peak derivative (0.332), and peak second derivative (0.332) were found to demonstrate relatively higher positive correlations, indicating their strong predictive power regarding tumor grade. In contrast, skewness (− 0.404) and median current (− 0.316) were found to exhibit significant negative correlations, suggesting that lower values of these features correspond to higher tumor grades. Similarly, for MUC-1, median current (− 0.541) and peak width at half height (− 0.443) were observed to show strong negative correlations, while the peak derivative (0.360) and kurtosis (0.295) displayed positive correlations, underlining their relevance in predicting tumor severity. These insights obtained from the correlation analysis are crucial for optimizing the predictive models for both biomarkers.

**Table 5:** Correlation coefficients of CV-derived features with tumor grades for biomarkers CA 15–3 and MUC-1

Feature	Correlation with CA 15–3	Correlation with MUC-1
Median current	− 0.3161	− 0.5413
Peak width at half height	− 0.0495	− 0.4434
Skewness	− 0.4048	− 0.2849
Negative peak current	− 0.0138	− 0.1729
Negative peak potential	0.2290	− 0.0997
Mean current	− 0.0620	− 0.0591
Half-peak potential	− 0.2592	− 0.0427
Peak second derivative	0.3321	− 0.0270
Standard deviation	0.1060	0.0976
Charge (Q)	0.0030	0.1402
Positive peak current	0.1499	0.1865
Positive peak potential	0.3297	0.2796
Kurtosis	0.4627	0.2955
Peak derivative	0.3327	0.3608

Following the correlation analysis, a feature importance analysis was conducted to further refine the understanding of the variables which most significantly impact the predictive models. A tree-based ensemble method was employed to estimate the importance of each feature in predicting tumor grades. The importance score assigned to each feature reflects its utility in enhancing the model's predictive accuracy, with higher scores indicating a greater influence on the model’s output. The results of the feature importance analysis are presented in Table 6. It enumerates the features along with their importance scores for each biomarker, offering a clear comparison of their relevance. For the MUC-1 biomarker, the most influential features, all with importance scores above 0.08, include charge (Q), mean current, peak second derivative, median current, kurtosis, skewness, and standard deviation. These features significantly contribute to the model, underscoring the diverse aspects of the CV signal that correlate with tumor severity. Similarly, for CA 15–3, features such as kurtosis, skewness, median current, peak second derivative, and positive peak current were identified as the most important, each surpassing the 0.08 importance threshold. This suggests that features related to the distribution’s shape (kurtosis and skewness) and specific peak characteristics (median current and peak second derivative) are crucial for predicting tumor grade from CA 15–3 data.

**Table 6:** Feature importance values for predicting tumor grades using CA 15–3 and MUC-1 biomarkers

Rank	Feature	Importance (CA 15–3)	Importance (MUC-1)
1	Kurtosis	0.1440	0.0868
2	Skewness	0.1062	0.0863
3	Median current	0.0884	0.0878
4	Peak second derivative	0.0880	0.0913
5	Positive peak current	0.0832	0.0739
6	Peak derivative	0.0744	0.0835
7	Negative peak current	0.0721	0.0720
8	Charge (Q)	0.0714	0.0985
9	Standard deviation	0.0678	0.0860
10	Mean current	0.0618	0.0922
11	Peak width at half height	0.0482	0.0590
12	Positive peak potential	0.0348	0.0353
13	Half-peak potential	0.0327	0.0335
14	Negative peak potential	0.0262	0.0132

The features selected for their high importance scores were utilized to train predictive models. The approach ensures that the models are not only accurate but also efficient, mitigating overfitting by excluding less informative features. By focusing on high-importance features, the models are closely aligned with the underlying biological and chemical phenomena evident in the CV data, thereby enhancing their clinical relevance and potential applicability.

Random Forest, XGBoost, LightGBM, and ANN algorithms are employed for developing a predictive model to determine the presence of tumor in canine samples. The dataset included CV signals from 18 samples (17 tumorous and one control). Each sample was represented by multiple features derived from CV analysis, as previously outlined in the feature importance section. The performance of each algorithm was evaluated based on its accuracy in correctly classifying the samples as tumorous or non-tumorous. The accuracies achieved were as follows:

- Random Forest: 98%
- XGBoost: 96%
- LightGBM: 95%
- ANN: 97%

These results indicate a high level of predictive accuracy, with Random Forest performing slightly better than the other models. Given the varied nature of these algorithms, ranging from ensemble tree-based methods to neural networks, this spread in performance underscores the robustness of the predictive modeling process, confirming that multiple advanced techniques are capable of high-precision tumor detection using electrochemical data.

The high accuracy rates achieved by these models emphasize their potential in clinical settings, where rapid and reliable tumor diagnostics are critical. The success of these algorithms in distinguishing between tumorous and nontumorous samples, even in a small dataset, suggests that the CV data contain significant diagnostic information that can be effectively extracted using ML algorithms. This capability could lead to improvements in noninvasive tumor diagnostics, offering faster, less costly, and more accessible options for veterinary medicine, and potentially adaptable methods for human healthcare.

Building on the successful detection of the presence of tumor in canine samples, the research advanced to the next phase: predicting the tumor grade based on CV data. Accurate tumor grading is crucial for determining the appropriate treatment strategies and making prognostic evaluations. Accurate training with cross-validation was conducted for each model to ensure that the findings were robust and not artifacts of the specific data split. For the CA 15–3 biomarker, the ANN model demonstrated the highest accuracy, achieving a 76% success rate in tumor grade predictions, which was notably superior compared to the other models. This result underscores the ANN’s ability to capture complex patterns and interactions within the CA 15–3 data, likely attributable to its layered structure and nonlinear processing capabilities. Conversely, the results for MUC-1, though slightly less effective overall, still demonstrated substantial predictive power, with the best-performing model (XGBoost) reaching an accuracy of 62%.

## Conclusion

In conclusion, it was determined that CA 15–3 in serum could be detected more sensitively rather than MUC-1 in tissue by biosensor and that the biosensor allowed the CA 15–3 tumor biomarker to be detected even at lower concentrations in serum samples in this study. The superior performance of CA 15–3 in predictive modeling, particularly with the ANN approach, suggests that CA 15–3 may possess more detectable or relevant characteristics to tumor progression stages than MUC-1. These findings could influence future directions, prompting further investigation into the molecular mechanisms underlying CA 15–3 biomarker’s capabilities. Moreover, the results indicate the potential in applying ML techniques to enhance the predictive accuracy of tumor diagnostics. By integrating these models into clinical settings, it may be possible to achieve more nuanced and accurate diagnosis which can be critical in tailoring treatment plans to individual patient needs effectively.

## Supplementary Information

Below is the link to the electronic supplementary material.

Supplementary file1 (DOCX 62 KB)
