# Supplementary material for: Enhancing electrochemical detection through machine learning-driven prediction for canine mammary tumor biomarker with green silver nanoparticles
Source: Anal Bioanal Chem. 2024 Jul 20;416(23):5071–88. doi: 10.1007/s00216-024-05444-0 (PMC11377509; doi:10.1007/s00216-024-05444-0)
Supplement: Supplementary file 1 — Supplementary file1 (DOCX 62 KB) [file 216_2024_5444_MOESM1_ESM.docx]

Supporting Information

**Enhancing Electrochemical Detection through Machine Learning-Driven Prediction for Canine Mammary Tumor Biomarker with Green Silver Nanoparticles**

a

b

c

d

e

f

g

h

**Figure S1:** CV responses with (a-d) CA 15-3 antibody from serum samples and (e-h) MUC-1 antibody from tissue homogenate samples.
